# Supplementary material for: Neuropathological correlates and genetic architecture of microglial activation in elderly human brain
Source: Nat Commun. 2019 Jan 24;10:409. doi: 10.1038/s41467-018-08279-3 (PMC6345810; doi:10.1038/s41467-018-08279-3)
Supplement: Supplementary file 1 — Supplementary Information [file 41467_2018_8279_MOESM1_ESM.pdf]

## Supplementary Information

# Neuropathological correlates and genetic architecture of microglial activation in elderly human brain

**Daniel Felsky PhD[1,2,†], Tina Roostaei MD[1,2], Kwangsik Nho PhD[3], Shannon L. Risacher PhD[3], Elizabeth M. Bradshaw PhD[1,2], Vlad Petyuk PhD[4], Julie A Schneider MD[5,6], Andrew Saykin MD[3], David A. Bennett MD[5,6], Philip L. De Jager MD[1,2]**

1. Center for Translational and Computational Neuroimmunology, Department of Neurology, Columbia University Medical Center, 630 West 168th Street, New York, NY 10032, USA.
2. Program in Population and Medical Genetics, Broad Institute of MIT and Harvard, 320 Charles Street, Cambridge, MA 02141, USA.
3. Indiana Alzheimer's Disease Center, Center for Neuroimaging, Department of Radiology and Imaging Sciences, Center for Computational Biology and Bioinformatics, Indiana University School of Medicine, 355 West 16th Street, Indianapolis, IN 46202, USA.
4. Pacific Northwest National Laboratory, Richland, WA, 99354, USA
5. Department of Neurology, Rush University Medical Center, 1653 West Congress Parkway, Chicago, IL 60612, USA.
6. Rush Alzheimer's Disease Center, Rush University Medical Center, 1653 West Congress Parkway, Chicago, IL 60612, USA.

[†]Corresponding Author

## Supplementary Methods

### Genetics and imputation

A total of 2 067 subjects had quality controlled genotype data. Genotypes were obtained on either the Illumina Human OmniExpress (*n*=381) or Affymetrix beadchip 6.0 (*n*=1 686) platform, and each group was pre-processed separately. Raw genotype data were cleaned and formatted using the Haplotype Reference Consortium (HRC) Imputation Preparation and Checking Tool (v4.2.6, http://www.well.ox.ac.uk/~wrayner/tools), then imputed using the Michigan Imputation Server (MIS)[1] and Eagle2[2] phasing software against the HRC reference panel (v1.1).[3] Following imputation, tri-allelic variants were removed and both groups of subjects were merged (subsequent genetic analyses co-varied for genotype platform). Output VCF format files from MIS were converted to PLINK dosage format using DosageConvertor (v1.0.3, http://genome.sph.umich.edu/wiki/DosageConvertor).

For the IMAS PET imaging sample, genotyping was performed using the Illumina HumanOmni-Express BeadChip. Standard quality control procedures were conducted as described previously.[4] The *TSPO* rs6971 genotype was imputed using IMPUTE (v2.2) and the 1000 Genomes (phase 1) reference panel. A posterior probability of 0.90 was used as a quality threshold for imputed genotypes. *APOE* (rs429358, rs7412) genotyping was carried out separately using standard protocols.

### Selected reaction monitoring proteomics

The panel of proteins was preselected from existing literature for their roles in neurodegenerative disease, microglial function, and inflammatory pathways. Samples were prepared for LC-SRM analysis using standard protocol. Briefly, on average ~20 mg of brain tissue from each subject was homogenized in denaturation buffer (8M urea, 50mM Tris-HCl pH 7.5, 10mM DTT, 1mM EDTA). Followed denaturation, 400µg protein aliquots were taken for further alkylation with iodoacetamide and digestion with trypsin. The digests were cleaned using solid phase extraction, followed readjustment of tryptic peptide digests concentration to 1µg/µL. 30µL aliquots were mixed with 30µL synthetic peptide mix. All liquid handling steps were performed in 96 well plate format using Epmotion 5075 TMX (Eppendorf) and Liquidator96 (Rainin).

All LC-SRM experiments were performed on a nano ACQUITY UPLC coupled to TSQ Vantage MS instrument, with 2 µL of sample injection for each measurement. A 0.1% FA in water and 0.1% in 90% ACN were used as buffer A and B, respectively. Peptide separations were performed by an ACQUITY UPLC BEH 1.7 µm C18 column (75µm i.d. × 25cm) at a flow rate 350nL/min using gradient of 0.5% of buffer B in 0-14.5min, 0.5-15% B in 14.5-15.0min, 15-40% B in 15-30min and 45-90% B in 30-32min. The heated capillary temperature and spray voltage was set at 350 °C and 2.4 kV, respectively. Both the Q1 and Q3 were set as 0.7 FWHM. The scan width of 0.002m/z and a dwell time of 10ms were used.

All the SRM data were analyzed by Skyline software.[5] All the data were manually inspected to ensure correct peak assignment and peak boundaries. The peak area ratios of endogenous light peptides and their heavy isotope-labeled internal standards (i.e., L/H peak area ratios) were then automatically calculated by the Skyline software and the best transition without matrix interference was used for accurate quantification. The peptide relative abundances were log2 transformed and centered at the median.

### Postmortem Neuropathology and microglial density quantification

All brains were examined by a board-certified neuropathologist blinded to clinical data. Brains were removed in a standard fashion as previously described.[6] After weighing, each brain was cut into 1cm coronal slabs using a Plexiglas jig. Slabs from one hemisphere, and slabs from the other hemisphere not designated for rapid freezing, were fixed for at least 3 days in 4% paraformaldehyde. We used defined landmarks to obtain at least two tissue blocks from each of the following regions: dorsolateral prefrontal cortex, middle and inferior temporal cortex, inferior parietal, hippocampus CA1/subiculum, entorhinal cortex proper, ventromedial caudate, and posterior putamen. Tissue blocks were processed, embedded in paraffin, cut into either 6 micron or 20µm sections, and mounted on glass slides. Neuropathologic diagnoses were made by a board-certified neuropathologist blinded to age and clinical data. Bielschowsky silver stain 6 micron sections were used to visualize neuritic plaques, diffuse plaques, and neurofibrillary tangles in the frontal, temporal, parietal, entorhinal, and hippocampal cortices, as previously described,[7] for the pathologic diagnosis of AD. A neuropathologic diagnosis of “no AD,” “low likelihood AD,” “intermediate likelihood AD,” or “high likelihood AD” was given based on semiquantitative estimates of neuritic plaque density as recommended by CERAD and the neurofibrillary tangle stage by Braak and Braak as recommended by the National Institute on Aging (NIA) - Reagan criteria. For analyses, the neuropathologic diagnosis of AD was considered absent if NIA-Reagan diagnosis was no or low and present if intermediate or high likelihood. The density of neuritic plaques, diffuse plaques and neurofibrillary tangles was characterized using Bielschowsky silver stain for visualization and a graticule

to count total number of each in a 1-mm$^2$ area (100 magnification) of highest density. Counts for each marker were completed for each of five regions (midfrontal cortex, middle temporal cortex, inferior parietal cortex, entorhinal cortex, and hippocampus CA1/subiculum) and then converted to standardized scores. Supplementary Table 9 describes the distributions of tau and amyloid neuropathologies in the pathologically confirmed AD and non-AD groups separately for both the entire available ROS/MAP cohort and the sample subset for which postmortem microglial density data were available.

Thirteen additional pathologies were measured in our cohort: Lewy bodies, macroscopic infarcts (acute, sub-acute, and chronic measured separately), microscopic infarcts (acute, sub-acute, and chronic measured separately), atherosclerosis, arteriolosclerosis, cerebral amyloid angiopathy (CAA), hippocampal sclerosis, neuronal loss in the substantia nigra,[8] and transactive response DNA-binding protein 43 kDa (TDP-43) proteinopathy. CAA was graded on a five-level scale (0 to 4) in four neocortical regions (mid-frontal, angular gyrus, inferior temporal gyrus, and calcarine cortex) and averaged to derive a CAA score, as previously described.[9] Chronic macroscopic and microscopic infarcts were each dichotomized as present or absent. Atherosclerosis was scored on a four-level severity scale, and arteriolosclerosis was measured on a four-level scale by small vessel pathologies in anterior basal ganglia.[10] Nigral, limbic, and neocortical Lewy bodies were dichotomized as present or absent, as identified using immunohistochemistry. Hippocampal sclerosis was recorded as either present or absent as evaluated with H&E stain. Pathological diagnosis of AD was given for cases with high or intermediate likelihood of AD per the modified National Institute of Aging–Reagan Institute criteria.[6] For a subset of participants ($n$ = 826), transactive response DNA-binding protein 43 kDa (TDP-43) proteinopathy was measured and categorized into four steps of severity as previously described:[11] no inclusions (stage 0), inclusions in amygdala only (stage I), inclusions in amygdala as well as entorhinal cortex and/or hippocampus CA1 (stage II), and inclusions in amygdala, neocortex, and entorhinal cortex and/or hippocampus CA1 (stage III). In addition, a semi-quantitative six-point scale for the severity of the TDP-43 cytoplasmic inclusions was rated as previously described ($n$=812).[12]

Immunohistochemistry for microglia was performed using an Automated Leica Bond immunostainer (Leica Microsystems Inc., Bannockborn IL) and anti-human HLA-DP, DQ, DR antibodies (clone CR3/43; DakoCytomation, Carpinteria CA; 1:100 dilution; catalog number MA1-25914) using standard Bond epitope retrieval and detection. An investigator blinded to the clinical and pathologic data, outlined the cortical or subcortical gray region of interest on each slide using a Microbrightfield Stereology System. The Stereo Investigator 8.0 software program was used to place a 1000 × 750μm sampling grid over the region and the program was engaged to sample 4.0% of the region with a 200 × 150μm counting frame at 400x magnification at interval grid intersection points. Using separate tags for stage I, II and III microglia, the operator marked the microglia at each intersection point. These counts were then upweighted by the stereology software to estimate total number of microglia (stage I, II, and III) in the defined area. Different stages of activation from least (stage I) to most (stage III) activated can be defined morphologically; when these cells become activated, their long fine processes contract and thicken and the cell body adopts a larger more rounded cellular conformation. Data from the two adjacent blocks of tissue (0.5 to 1.0 cm apart) were averaged to obtain composite average densities of microglia in each region.

**Longitudinal cognitive decline metric**

All subjects were administered 17 cognitive tests annually spanning five cognitive domains. The full list of tests is provided in Supplementary Table 10. Rates of cognitive decline for each domain were calculated per subject using general linear mixed models of cognitive scores over time, co-varying for age at baseline, years of education, and sex, as described.[13]

**[$^{11}$C]-PBR28 PET image acquisition and analysis**

An anatomic 3D magnetization-prepared rapid-acquisition gradient-echo (MP-RAGE) MR imaging sequence was acquired on a 3T Tim Trio (Siemens). [$^{11}$C]-PBR28 was synthesized as described previously.[14] Dynamic PET scans (HR+; Siemens) were initiated with injection of approximately 555MBq of [$^{11}$C]-PBR28 (details previously published).[14] Data were acquired for 90 min, with static images generated from data between the 35-90 min interval. PET imaging data were motion-corrected and normalized to Montreal Neurological Institute (MNI) coordinates using previously described methods.[15] Standardized uptake value (SUV) images were created by normalizing each voxel by the injected dose of [$^{11}$C]-PBR28 per total body weight. Statistical models of the effect of rs2997325 genotype included *TSPO* rs6971 (technical covariate to control for altered TSPO binding characteristics[14]), *APOE* ε4 status, age at study entry, and sex as co-variates.

For these analyses, we used three regions of interest (ROIs) for each hemisphere separately, resulting in six brain regions. First, we performed linear regression analyses using the six ROI values separately and identified an association in the left entorhinal cortex (uncorrected $p$-value < 0.05). As six ROI values were strongly correlated and the sample size is moderate for genetic analysis of this

type ($n$=27), we carried out a multivariate analysis by performing genetic association analysis using six phenotypes simultaneously to minimize the number of test performed and increase the statistical power. We used the SCOPA (Software for Correlated Phenotype Analysis) program[16] to perform an aggregation of genetic analyses for our multiple correlated phenotypes.

### PAM metric validation using logistic regression

The 'rms' R package was used to perform logistic regression model performance and validation. The main challenge presented by these data was the zero-inflated, non-normal distribution of stage III microglial densities found across regions (Figure 1). This in turn resulted in a zero-inflation of the PAM measures, as stage III density is found in the numerator of the PAM equation. Therefore, the effect of a PAM term evaluated continuously within a logistic model of pathological Alzheimer's disease (pathoAD) status may only be representing an underlying binary effect, whereby the difference in probability of having pathoAD is driven by individuals with none vs. some stage III microglia. To test this, we calculated four versions of each cortical PAM score (as the subcortical stage III microglia showed no association with pathoAD): 1) 'continuous': the full range of PAM as a numerical variable, 2) 'med. split': the continuous measure binarized by coding all values less than the median as 0 and all those equal or greater than the median as 1, 3) 'zero vs. else': the continuous measure binarized by coding all values of 0 as 0, and anything larger than zero as 1, and 4) 'no zeros': the continuous measure but only taking a subset of subjects in the dataset by excluding all subjects with a value of 0. These transformations are represented graphically on the midfrontal PAM measure in Supplementary Figure 2a.

For each of these transformations, we tested a series of models with different covariates and evaluated area under the receiver operating characteristics curve (AUC) as our metric of model performance. The results of all tested models are found in Supplementary Table 1. In addition to testing for improvements in model performance, we examined diagnostic plots of influence and leverage, as well as residual distributions, to confirm that regression assumptions were not violated. A bootstrapping method known as 0.632+, which accounts for the probability of resampling, was used for further assessment of model validity and generalizability (1 000 iterations). Bootstrapping allows for the calculation of model optimism (overfitting) and thus provides estimates of calibrated AUCs.

Following this comprehensive validation procedure, we found that the continuous measure of PAM (transformation 1) outperformed the other three transformations both before and after bootstrapping, particularly for PAM measured in the inferior temporal cortex (Supplementary Figure 2b). The 'med. split' transformation performed similarly to the 'continuous' measure for PAM in the midfrontal cortex, but still with less accuracy. Also, while the inclusion of the 'zero vs. else' PAM term did improve model accuracy over the *APOE* + covariates only model, these models performed substantially worse than the 'med. split' and 'continuous' measures in both inferior temporal and midfrontal cortex, suggesting that the effect of PAM is driven both by the presence vs. absence of stage III microglia and the continuous spectrum of relative activation in those with non-zero stage III counts. Note that AUC values differ between the 'no zeros' models and the others even where the PAM term is not included; this is due to the difference in sample size in this analysis where subjects with PAM=0 were removed. Also note minor variation in the bootstrapped model AUCs where no PAM term is present; this is due to the (pseudo)random nature of the bootstrap procedure and is expected.

### Hierarchical clustering of microglial density and PAM phenotypes

Pearson correlations were calculated between each pair of microglial phenotypes. Statistical significance was determined using a false discovery rate threshold of $q$=0.05. To order phenotypes within the correlation matrix, hierarchical clustering was then performed using the corrMatOrder() function in the R 'corrplot' package. To objectively determine the optimal number of clusters to impose on the data, the 'NbClust' package was used to simultaneously evaluate 25 metrics of best fit for cluster number.[17] Through majority vote across metrics, three clusters provided an optimal solution (Supplementary Figure 1).

### Regression of PAM against pathological AD diagnosis and neuropathologies

Regression modeling was performed using the 'rms' and 'MASS' packages in R (v3.3.3). Each PAM measure was first tested for effects on pathological diagnosis of AD using logistic regression in a full model, co-varying for age at death, sex, postmortem interval (PMI), genotype batch, top three EIGENSTRAT principal components (PCs) (to control for fine population stratification), and *APOE* ε4 status. For iterative re-weighted least squares regression, the Huber psi function[18] was used for coefficient estimation. The .632+ bootstrap method was chosen for model validation as it takes into account the probability of resampling observations and thus outperforms traditional cross-validation and bootstrapping algorithms.[19]

## Causal mediation modeling

We assumed *a priori* the canonical cascade of AD etiopathology where the influence of beta-amyloid on cognitive decline lies upstream of tau hyperphosphorylation (measured as paired helical filament (PHF) tau); this model has been shown to be statistically valid in the ROS/MAP cohort using mediation modeling.[20] Based on this assumption, we tested only models where amyloid effects are upstream of tau, but alternated the role of PAM between effector and mediator to ascertain its likely position within the pathological cascade. To estimate confidence intervals for average indirect, direct, and total effects, 1 000 Monte Carlo draws were used for nonparametric bootstrapping.

## Genome-wide association analysis

Only high quality variants (imputed info score > 0.8) were included in analyses. All models co-varied for age at death, postmortem interval, sex, genotype batch, and the first three EIGENSTRAT[21] principal components. Significance thresholds of $p<2.5\times10^{-8}$ and $p<1.0\times10^{-5}$ were deemed genome-wide significant (corrected for two GWAS) and suggestive, respectfully. To contrast results from our GWAS, overlap was evaluated using the 'Q-value' Bioconductor package (https://github.com/StoreyLab/qvalue),[22] where true positive rate is estimated and overlapping proportions of SNPs between GWAS are tested.

For post-processing of each set of GWAS results, we used the recently released Functional Mapping and Annotation of Genome-Wide Association Studies (FUMAGWAS; http://fuma.ctglab.nl/) platform.[23] This platform performs a series of state-of-the art SNP- and gene-level functional mapping steps to distill and explore the biological relevance of GWAS findings based on the most recent bioinformatics database updates. As of Feb 21, 2018 (after which our results were processed) these databases include the latest eQTL data releases from the Genotype-Tissue Expression project (GTEx version 7; https://www.gtexportal.org/home/),[24] the Multiple Tissue Human Expression Resource (MuTHER; http://www.muther.ac.uk/Data.html), the Brain eQTL Almanac from the UK Brain Expression Consortium (BRAINEAC; http://www.braineac.org/),[25] the CommonMind Consortium (https://www.synapse.org/cmc),[26] and the ROS/MAP xQTL server (http://mostafavilab.stat.ubc.ca/xQTLServe/).[27] SNP2GENE mapping was performed using the following parameters: lead SNPs defined at $p<1\times10^{-5}$, clumping minimum $r^2$ for LD in risk loci of 0.6 with accompanying association $p<0.05$, inclusion of all 1000 Genomes Phase 3[28] reference SNPs in LD calculations, 250kb window for merging risk loci, and maximum distance for physical gene mapping of 10kb. Gene mapping using eQTL data incorporated all tissue type data from all aforementioned databases. All other parameters were chosen as default. Additionally, independent SNP signals within each identified risk locus were assessed for Combined Annotation Dependent Depletion (CADD v1.3) score,[29] RegulomeDB (v1.1; http://www.regulomedb.org/) score,[30] and occurrence in the Catalog of published GWAS (https://www.ebi.ac.uk/gwas/).[31] Genes mapped to risk loci based on combined criteria were carried forward for GENE2FUNC analysis, also implemented using the FUMAGWAS platform.

Tissue expression specificity for each mapped gene was evaluated using GTEx v7 across 53 tissue types and displayed as average expression per label (log2 transformed). Tissue-specific enrichment of the set of mapped genes for each GWAS was evaluated using hypergeometric tests based on pre-computed differentially expressed gene (DEG) t-tests, where Bonferroni *p*-values of $p<0.05$ or absolute log fold change $\geq 0.58$ constituted significant differential expression. Gene set enrichment analysis was performed using Molecular Signatures Database[32] (MSigDB v5.2) collections, WikiPathways[33] (curated version 20161010), and the GWAS catalog (reported genes, version e91; 20180206) (number of unique background genes=34 748). Finally, the DrugBank database[34] (version 5.0.11; https://www.drugbank.ca/) was used to identify drugs targeting mapped genes for potential therapeutic translation.

## Mendelian randomization using summary statistics

For each cortical PAM GWAS, summary statistics were used to generate a set of scores (one score per subject, per GWAS), which comprise linear combinations of PAM-associated alleles weighted by their effect coefficients. The PRSice pipeline performs SNP clumping based on the original ROS/MAP imputed genetic data to eliminate score bias due to linkage disequilibrium. Gene variants and weights can then be applied to external GWAS summary statistic datasets to calculate estimated effect coefficients for the group of PAM-associated variants on each external GWAS trait for a range of 10 000 *p*-value thresholds (lower limit $p = 0$, upper limit $p = 0.5$, increment = $5.0\times10^{-5}$). This method was repeated in the reverse direction (i.e. calculating scores from the published GWAS and estimating effect coefficients for the score on each PAM trait) to assess causal relationships of our selected traits on microglial activation in the aging brain.

Our results pinpoint educational attainment as an important phenotype related to the genetic architecture of microglial activation. The published study from which educational attainment GWAS statistics were extracted is Okbay et al., 2016.[35] The phenotype was calculated based on a standard in the 1997 International Standard Classification of Education (ISCED) of the United Nations Educational, Scientific and Cultural Organization. Due to the diversity of study settings from which meta-analyzed samples were collected, it was necessary for authors to map each major educational qualification that it is possible to attain in a specific country into one of seven harmonized ISCED categories. Their main outcome variable was then imputed as a years-of-education equivalent for each ISCED category.

## Supplementary Figures

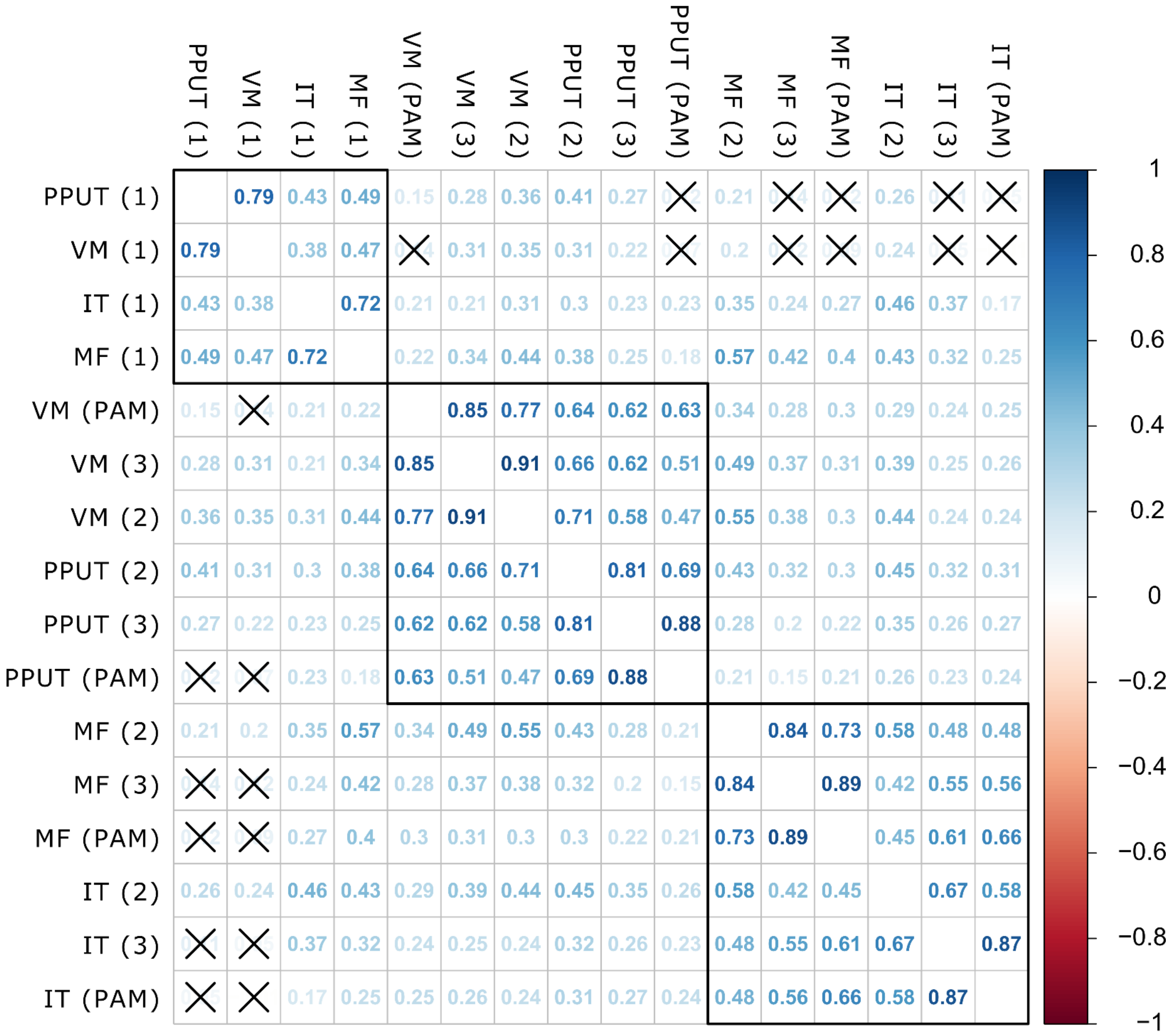


***Supplementary Figure 1*. Spearman correlations of all microglial phenotypes across four brain regions**. Statistical significance was determined using a false discovery rate (FDR) threshold of *q*=0.05 (the X symbols mark correlations that were not statistically significant according to two-sided FDR-corrected *p*-value). Phenotypes were ordered by hierarchical clustering. IT = inferior temporal cortex; MF = midfrontal cortex; PPUT = posterior putamen; VM = verntal medial caudate.

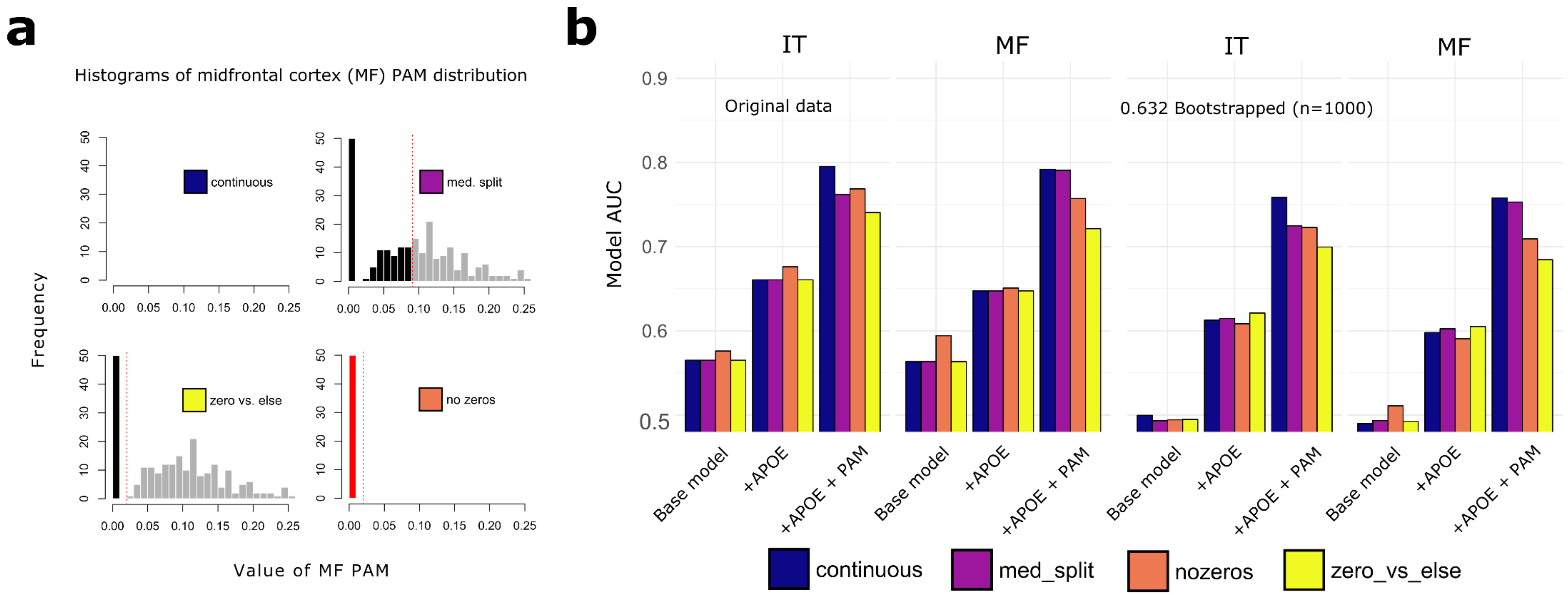


***Supplementary Figure 2.*** **Results of model validation for PAM phenotype.** A) distribution of PAM is shown for midfrontal cortex (MF). B) model performance (indicated by area under the curve (AUC) of receiver operating characteristic curves (AUC)) is shown for the original dataset as well as for an average of AUCs over 1 000 bootstrapped samples using the .632+ method. The model term indicated as "APOE" is a variable of *APOE* ε4 genotype status (dichotomous, as positive or negative). IT = inferior temporal cortex. The base model includes co-variates sex, age at death, and postmortem interval.

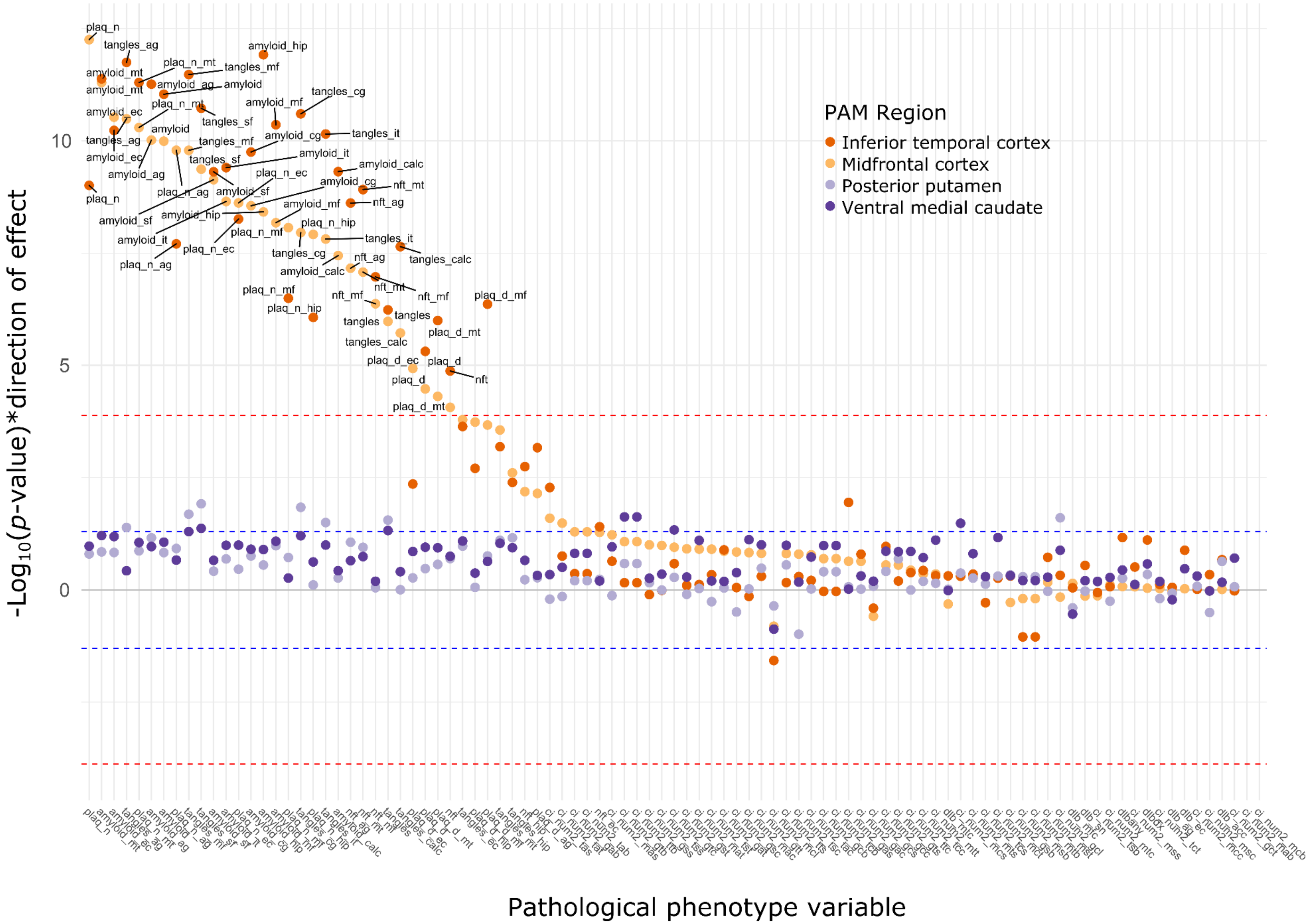


***Supplementary Figure 3*****. Robust regression testing associations of PAM with regional neuropathologies**. The y-axis represents $-\log_{10}$(p-values) for the PAM measure term in regression models, weighted by the sign of the effect coefficent (1 or -1). *P*-values are two-sided. The list includes the original 18 types of tested neuropathologies, but broekn down into their original component observations rather than the brain-wide aggregates presented in Figure 3. Only amyloid and tau-related pathoogies were significantly associated with any PAM measure. For a full list of variable names and summaries, see https://www.radc.rush.edu/docs/var/variables.htm.

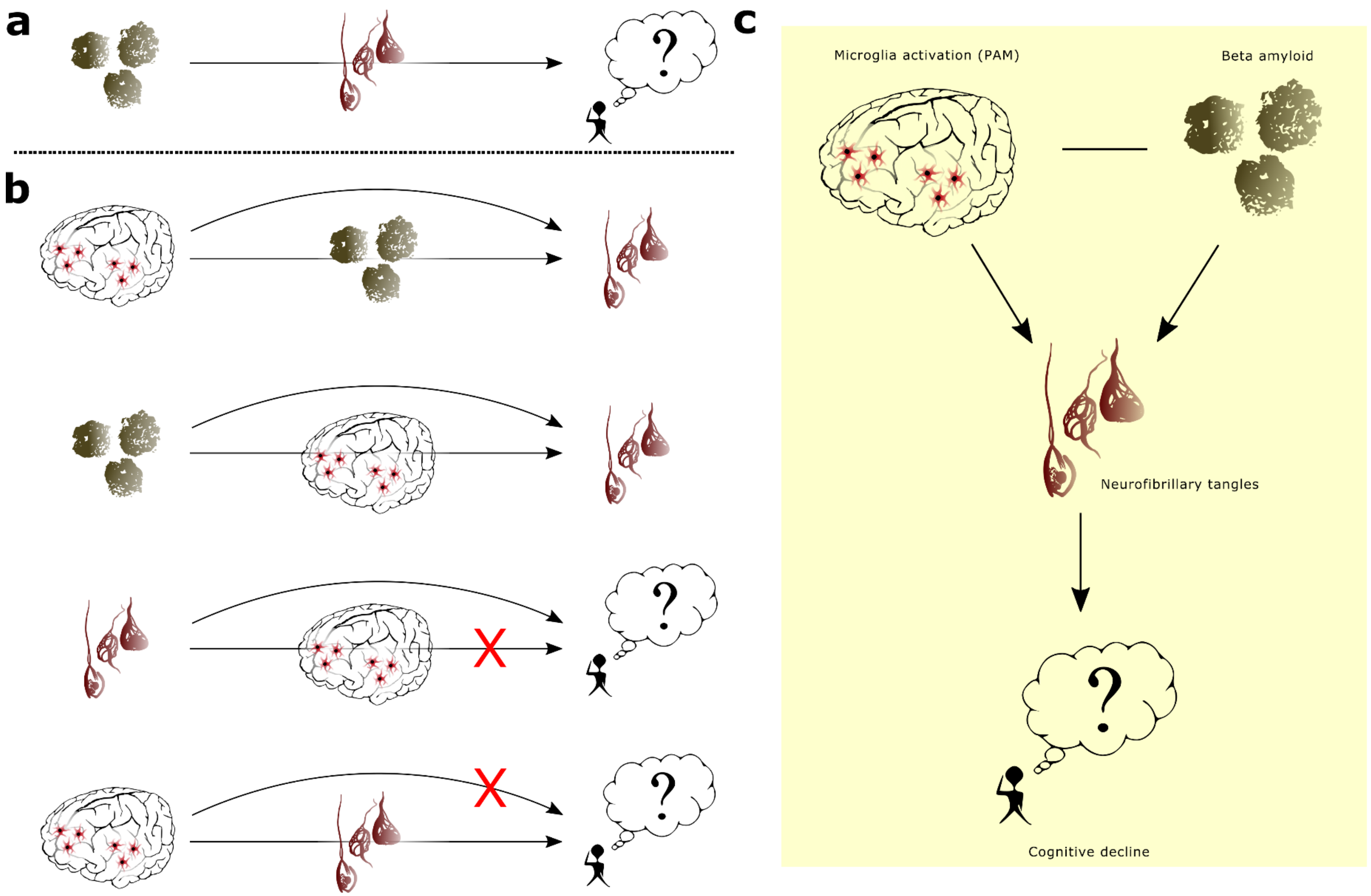


***Supplementary Figure 4*. Causal mediation analysis for direct and indirect effects of PAM**. A) illustration of our assumed a priori model of the canonical AD pathological cascade, where beta-amyloid causes tau hyperphosphorylation, which causes downstream cognitive decline. B) Results from eight mediation models, where each black line represents a direct or mediated effect, and the red X denotes a non-significant effect (two-sided $p>0.05$). C) summary figure showing the proposed sequence of events. Supplementary Table 2 shows full results for each model tested ($n=225$ for midfrontal cortex, $n=205$ for inferior temporal cortex; mediation effects were the same for MF and IT). Beta amyloid figure adapted from Darvesh, Hopkins & Geula (2003) https://www.nature.com/articles/nrn1035#rightslink. Neurofibrillary tangles figure adapted from Alzheimer (1911) Ueber eigenartige Krankheitsfaelle des spaeteren Alters (https://doi.org/10.1177/0957154X9100200506).

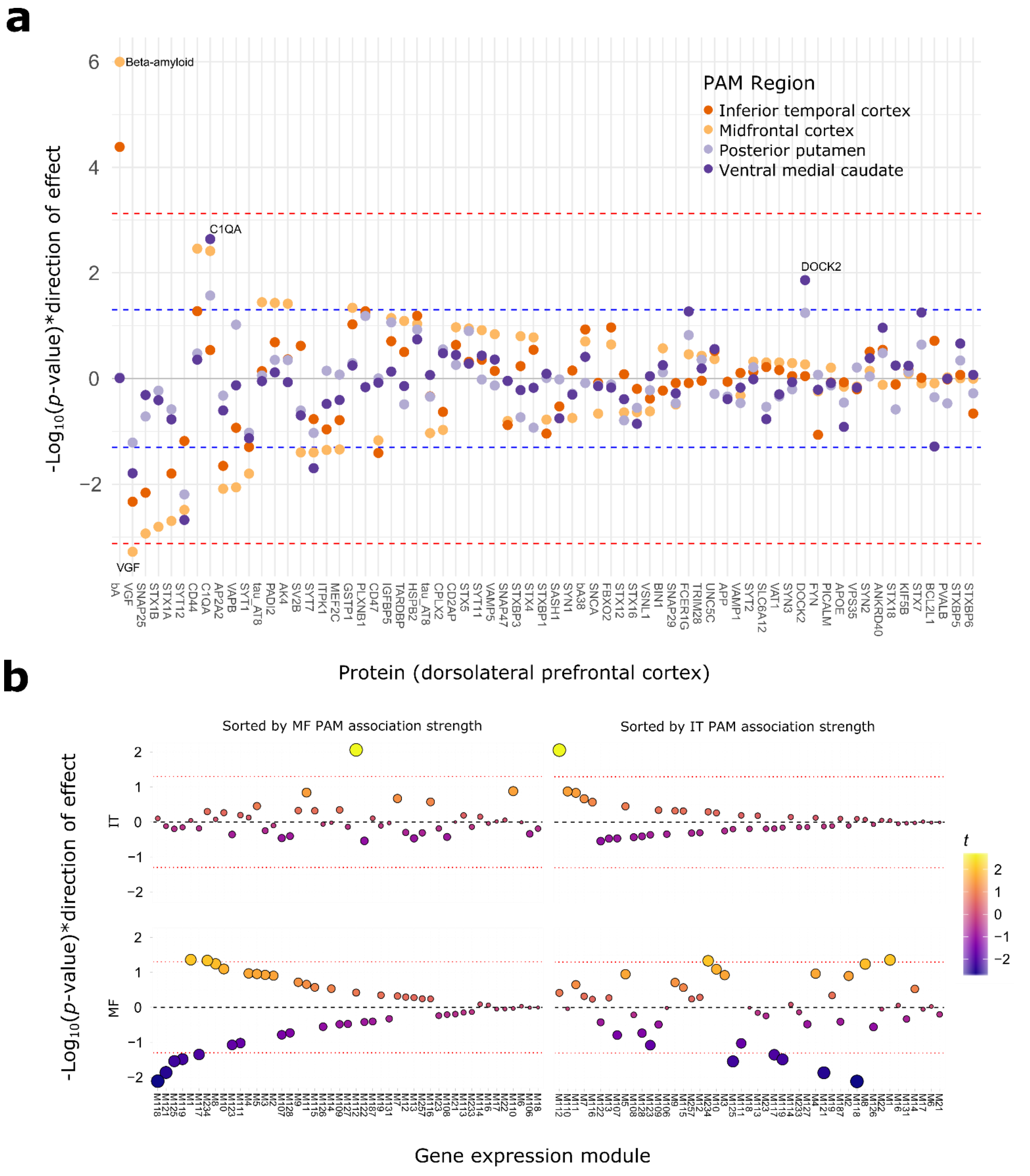


***Supplementary Figure 5*. PAM associations with proteome and transcriptome**. A) Effects of PAM measures on protein levels in postmortem dorsolateral prefrontal cortex (DLPFC), assessed using robust regression (*n*=164-187). B) Effects of cortical PAM measures (inferior temporal cortex (IT) on top, midfrontal cortex (MF) on bottom) on the expression levels of 47 gene modules, derived from ~13 000 expressed genes in the DLPFC (*n*=100-102). The left side plots are sorted from left to right by decreasing effect of MF PAM on gene module expression. The right side plots are sorted from left to right by decreasing effect of IT PAM on gene module expression (color spectrum corresponds to T statistic). Both y-axes represent $-\log_{10}$(*p*-values) for the PAM measure term in robust regression models, weighted by the sign of the effect coefficient (1 or -1). *P*-values are two-sided.

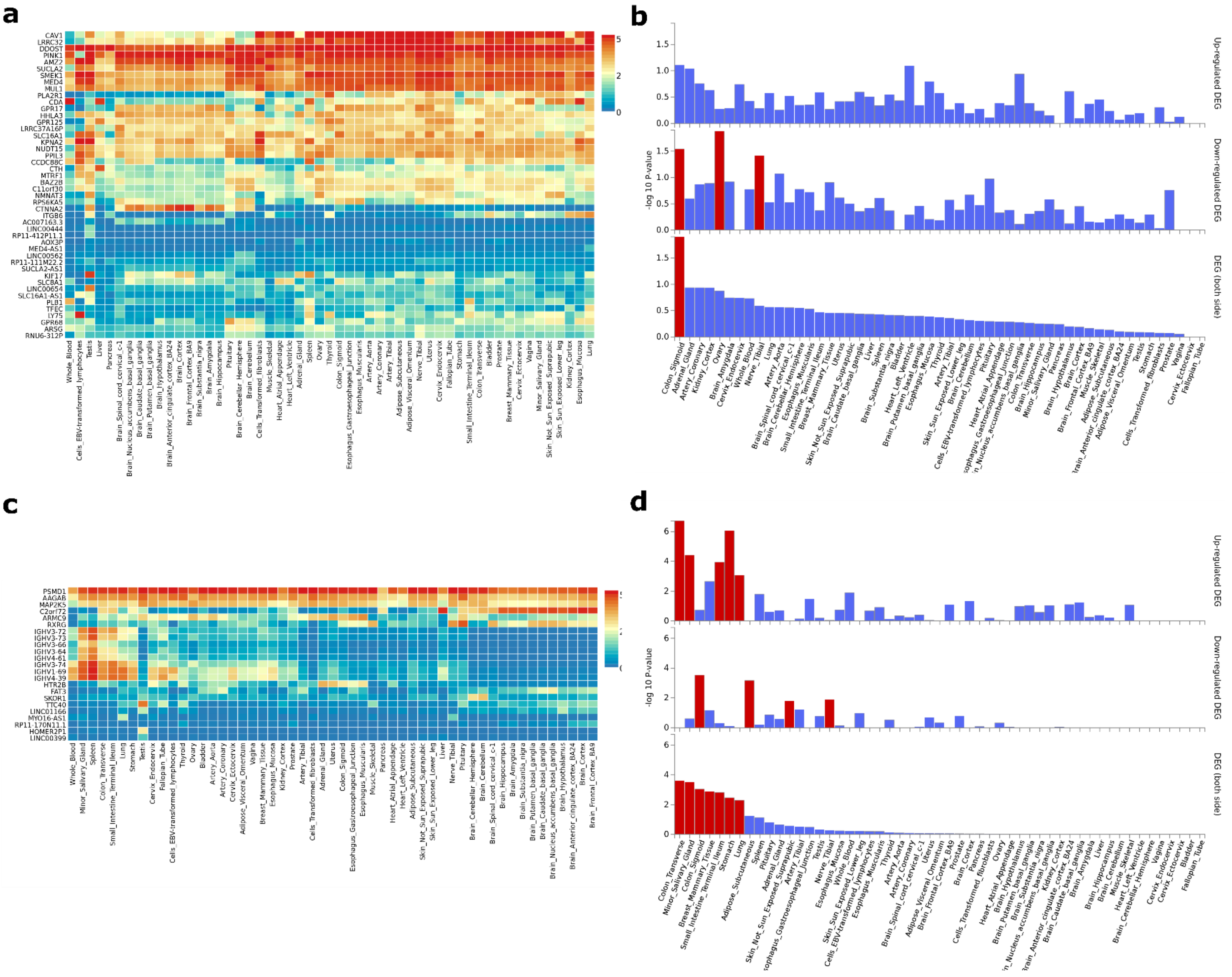


***Supplementary Figure 6*. Tissue enrichment analyses in 53 tissue types (GTEx v7) for gene sets mapped from cortical PAM GWAS**. Heat maps showing average expression of each gene mapped to significant and suggestive loci in the A) inferior temporal cortex (IT) and C) midfrontal cortex (MF) PAM GWAS (y-axis) in each tissue type (x-axis). Color scale indicates expression level in $\log_2$(TPM) where red = higher expression and blue = lower expression. Corresponding differential gene expression analyses for each tissue type according to mapped gene sets from the B) IT and D) MF GWAS. The top, middle, and bottom plots in panels B and D correspond to up-regulated (one-sided), down-regulated (one-sided), and differentially-regulated (two-sided) analyses, respectively. The y-axes are $-\log_{10}$($p$-values) and the bar colors represent statistical significance corrected for multiple testing (red is significant and blue is not). Tissue types (x-axis) are ordered from left to right by decreasing significance in differential expression (two-sided) analyses (bottom plots of panels B and D).

## Supplementary References


1. Das, S. *et al.* Next-generation genotype imputation service and methods. *Nat. Genet.* **48,** 1284–1287 (2016).
2. Loh, P.-R. *et al.* Reference-based phasing using the Haplotype Reference Consortium panel. *Nat. Genet.* **48,** 1443–1448 (2016).
3. McCarthy, S. *et al.* A reference panel of 64,976 haplotypes for genotype imputation. *Nat. Genet.* **48,** 1279–1283 (2016).
4. Kim, S. *et al.* Genome-wide association study of CSF biomarkers Abeta1-42, t-tau, and p-tau181p in the ADNI cohort. *Neurology* **76,** 69–79 (2011).
5. MacLean, B. *et al.* Skyline: an open source document editor for creating and analyzing targeted proteomics experiments. *Bioinforma. Oxf. Engl.* **26,** 966–968 (2010).
6. Bennett, D. A. *et al.* Neuropathology of older persons without cognitive impairment from two community-based studies. *Neurology* **66,** 1837–1844 (2006).
7. Schneider, J. A., Wilson, R. S., Bienias, J. L., Evans, D. A. & Bennett, D. A. Cerebral infarctions and the likelihood of dementia from Alzheimer disease pathology. *Neurology* **62,** 1148–1155 (2004).
8. Buchman, A. S. *et al.* Nigral Pathology and Parkinsonian Signs in Elders without Parkinson's Disease. *Ann. Neurol.* **71,** 258–266 (2012).
9. Boyle, P. A. *et al.* Cerebral amyloid angiopathy and cognitive outcomes in community-based older persons. *Neurology* **85,** 1930–1936 (2015).
10. Yip, A. G. *et al.* APOE, vascular pathology, and the AD brain. *Neurology* **65,** 259–265 (2005).
11. Yu, L. *et al.* The TMEM106B locus and TDP-43 pathology in older persons without FTLD. *Neurology* **84,** 927–934 (2015).
12. Wilson, R. S. *et al.* TDP-43 Pathology, Cognitive Decline, and Dementia in Old Age. *JAMA Neurol.* **70,** 1418–1424 (2013).
13. De Jager, P. L. *et al.* A genome-wide scan for common variants affecting the rate of age-related cognitive decline. *Neurobiol. Aging* **33,** 1017.e1–15 (2012).
14. Yoder, K. K. *et al.* Influence of TSPO genotype on 11C-PBR28 standardized uptake values. *J. Nucl. Med. Off. Publ. Soc. Nucl. Med.* **54,** 1320–1322 (2013).
15. Yoder, K. K. *et al.* Test-retest variability of [$^{11}$C]raclopride-binding potential in nontreatment-seeking alcoholics. *Synap. N. Y. N* **65,** 553–561 (2011).
16. Mägi, R. *et al.* SCOPA and META-SCOPA: software for the analysis and aggregation of genome-wide association studies of multiple correlated phenotypes. *BMC Bioinformatics* **18,** 25 (2017).
17. NbClust: An R Package for Determining the Relevant Number of Clusters in a Data Set | Charrad | Journal of Statistical Software. doi:10.18637/jss.v061.i06
18. Huber, P. J. *Robust statistics*. (1981).

19. Efron, B. & Tibshirani, R. Improvements on Cross-Validation: The .632+ Bootstrap Method. *J. Am. Stat. Assoc.* **92,** 548–560 (1997).
20. Bennett, D. A., Schneider, J. A., Wilson, R. S., Bienias, J. L. & Arnold, S. E. Neurofibrillary tangles mediate the association of amyloid load with clinical Alzheimer disease and level of cognitive function. *Arch. Neurol.* **61,** 378–384 (2004).
21. Price, A. L. *et al.* Principal components analysis corrects for stratification in genome-wide association studies. *Nat. Genet.* **38,** 904–909 (2006).
22. Storey, J. D. & Tibshirani, R. Statistical significance for genomewide studies. *Proc. Natl. Acad. Sci.* **100,** 9440–9445 (2003).
23. Watanabe, K., Taskesen, E., Bochoven, A. & Posthuma, D. Functional mapping and annotation of genetic associations with FUMA. *Nat. Commun.* **8,** 1826 (2017).
24. GTEx Consortium *et al.* Genetic effects on gene expression across human tissues. *Nature* **550,** 204–213 (2017).
25. Ramasamy, A. *et al.* Genetic variability in the regulation of gene expression in ten regions of the human brain. *Nat. Neurosci.* **17,** 1418–1428 (2014).
26. Fromer, M. *et al.* Gene expression elucidates functional impact of polygenic risk for schizophrenia. *Nat. Neurosci.* **19,** 1442–1453 (2016).
27. Ng, B. *et al.* An xQTL map integrates the genetic architecture of the human brain's transcriptome and epigenome. *Nat. Neurosci.* **20,** 1418 (2017).
28. 1000 Genomes Project Consortium *et al.* A global reference for human genetic variation. *Nature* **526,** 68–74 (2015).
29. Kircher, M. *et al.* A general framework for estimating the relative pathogenicity of human genetic variants. *Nat. Genet.* **46,** 310–315 (2014).
30. Boyle, A. P. *et al.* Annotation of functional variation in personal genomes using RegulomeDB. *Genome Res.* **22,** 1790–1797 (2012).
31. MacArthur, J. *et al.* The new NHGRI-EBI Catalog of published genome-wide association studies (GWAS Catalog). *Nucleic Acids Res.* **45,** D896–D901 (2017).
32. Liberzon, A. *et al.* Molecular signatures database (MSigDB) 3.0. *Bioinformatics* **27,** 1739–1740 (2011).
33. Kutmon, M. *et al.* WikiPathways: capturing the full diversity of pathway knowledge. *Nucleic Acids Res.* **44,** D488-494 (2016).
34. Wishart, D. S. *et al.* DrugBank: a knowledgebase for drugs, drug actions and drug targets. *Nucleic Acids Res.* **36,** D901-906 (2008).
35. Okbay, A. *et al.* Genome-wide association study identifies 74 loci associated with educational attainment. *Nature* **533,** 539–542 (2016).
36. Olah, M. *et al.* A transcriptomic atlas of aged human microglia. *Nat. Commun.* **9,** 539 (2018).
