# Supplementary material for: Neuropathological correlates and genetic architecture of microglial activation in elderly human brain
Source: Nat Commun. 2019 Jan 24;10:409. doi: 10.1038/s41467-018-08279-3 (PMC6345810; doi:10.1038/s41467-018-08279-3)
Supplement: Supplementary file 3 — Description of Additional Supplementary Information [file 41467_2018_8279_MOESM3_ESM.pdf]

Description of Additional Supplementary Files

File Name: Supplementary Data 1
Description: Results of model validation procedures comparing microglial phenotypes and transformations. Note: covariates (covs.) included in all models were age at death, sex, postmortem interval, and top three genomic principal components (population stratification). Odds ratios for continuous microglial phenotype terms ('continuous' and 'no zeros') were calculated as that corresponding to an increase of one interquartile range (25th percentile to 75th percentile). Optimal thresholds for calculations of sensitivity and specificity were determined using Youden's J statistic. AUC = area under the curve (receiver operating characteristics (ROC) curve); IT = inferior temporal cortex; MF = midfrontal cortex; PPUT = posterior putamen; VM = ventral medial caudate.

File Name: Supplementary Data 2
Description: Results for individual mediation models. Note: Sig. is denoted as * if $p<0.05$ and NS if $p>0.05$. Effects are reported from causal mediation analyses using nonparametric bootstrapped confidence intervals (1000 iterations). PHF tau = paired helical filament tau; Cog dec. = global cognitive decline (longitudinal); Aβ = total beta amyloid burden; MF = midfrontal cortex; IT = inferior temporal cortex. n=225 for MF and n=205 for IT.

File Name: Supplementary Data 3
Description: Full results of proteomic analyses for each PAM region. Note: modeling was performed using iterative re-weighted least squares regression (with Huber psi function determining M-estimator) including the following co-variates: age at death, sex, protein plate, postmortem interval, and top three genomic principl components (population stratification). IT = inferior temporal cortex; MF = midfrontal cortex; PPUT = posterior putamen; VM = ventral medial caudate. Colors indicate p-values less than 0.05, with darker color indicating lower p-value.

File Name: Supplementary Data 4
Description: Full PAM GWAS results showing effects of all lead SNPs in both MF and IT regions. Note: Final 10 columns show the results for each association locus lead SNP in both GWAS, to compare relative effects on microglial activation in both cortical regions. IT = inferior temporal cortex; MF = midfrontal cortex.

File Name: Supplementary Data 5
Description: List of PAM GWAS mapped genes, attributes, and eQTL tissues. Note: Full annotation of column names can be found on the FUMAGWAS tutorial page (http://fuma.ctglab.nl/tutorial#outputs).

File Name: Supplementary Data 6
Description: Results of gene set enrichment analyses performed for IT and MF PAM GWAS.

File Name: Supplementary Data 7
Description: List of 29 published GWAS summary statistics datasets used for polygenic scoring analyses.

File Name: Supplementary Data 8
Description: Drug targets for PAM GWAS-implicated genes from DrugBank (https://www.drugbank.ca/).

File Name: Supplementary Data 9
Description: Distribution of amyloid and tau neuropathologies in pathologically confirmed Alzheimer's disease (pathoAD) and non-Alzheimer's disease (non-pathoAD) subjects.

File Name: Supplementary Data 10
Description: List of cognitive tests administered to Religious Orders Study and Memory and Aging Project participants.
