# Supplementary material for: Neuropathological correlates and genetic architecture of microglial activation in elderly human brain
Source: Nat Commun. 2019 Jan 24;10:409. doi: 10.1038/s41467-018-08279-3 (PMC6345810; doi:10.1038/s41467-018-08279-3)
Supplement: Supplementary file 14 — Reporting Summary [file 41467_2018_8279_MOESM14_ESM.pdf]

nature research

Corresponding author(s): Philip L. De Jager, Daniel Felsky

Reporting Summary

Nature Research wishes to improve the reproducibility of the work that we publish. This form provides structure for consistency and transparency in reporting. For further information on Nature Research policies, see Authors & Referees and the Editorial Policy Checklist.

Statistical parameters

When statistical analyses are reported, confirm that the following items are present in the relevant location (e.g. figure legend, table legend, main text, or Methods section).

n/a	Confirmed	
☐	☒	The exact sample size (*n*) for each experimental group/condition, given as a discrete number and unit of measurement
☐	☒	An indication of whether measurements were taken from distinct samples or whether the same sample was measured repeatedly
☐	☒	The statistical test(s) used AND whether they are one- or two-sided<br>*Only common tests should be described solely by name; describe more complex techniques in the Methods section.*
☐	☒	A description of all covariates tested
☐	☒	A description of any assumptions or corrections, such as tests of normality and adjustment for multiple comparisons
☐	☒	A full description of the statistics including central tendency (e.g. means) or other basic estimates (e.g. regression coefficient) AND variation (e.g. standard deviation) or associated estimates of uncertainty (e.g. confidence intervals)
☐	☒	For null hypothesis testing, the test statistic (e.g. *F*, *t*, *r*) with confidence intervals, effect sizes, degrees of freedom and *P* value noted<br>*Give P values as exact values whenever suitable.*
☒	☐	For Bayesian analysis, information on the choice of priors and Markov chain Monte Carlo settings
☐	☒	For hierarchical and complex designs, identification of the appropriate level for tests and full reporting of outcomes
☐	☒	Estimates of effect sizes (e.g. Cohen's *d*, Pearson's *r*), indicating how they were calculated
☐	☒	Clearly defined error bars<br>*State explicitly what error bars represent (e.g. SD, SE, CI)*

Our web collection on statistics for biologists may be useful.

Software and code

Policy information about availability of computer code

Data collection	Stereo Investigator Software (v8.0) was used for sampling of tissue grids for microglial cell counting.
Data analysis	R Statistical Software (v3.3.3) and PLINK (v1.90b) were the main software packages used for analysis of neuropathological and genomic data. Further, Statistical Parametric Mapping (SPM8) and Freesurfer (v5.1), and Software for Correlated Phenotype Analysis (SCOPA) were used to analyze imaging data. PRSice (v1.25) was used to perform polygenic analyses, and the online FUMAGWAS server was used for post-GWAS enrichment analyses. All programs, versions, and relevant R packages have been explicitly stated in the manuscript to ensure reproducibility.

For manuscripts utilizing custom algorithms or software that are central to the research but not yet described in published literature, software must be made available to editors/reviewers upon request. We strongly encourage code deposition in a community repository (e.g. GitHub). See the Nature Research guidelines for submitting code & software for further information.

April 2018

Data

Policy information about availability of data

All manuscripts must include a data availability statement. This statement should provide the following information, where applicable:

- Accession codes, unique identifiers, or web links for publicly available datasets
- A list of figures that have associated raw data
- A description of any restrictions on data availability

Access to ROS/MAP data used in the preparation of this manuscript can be applied for at the Rush Alzheimer's Disease Center Resource Sharing Hub (https://www.radc.rush.edu/).

Field-specific reporting

Please select the best fit for your research. If you are not sure, read the appropriate sections before making your selection.

☒ Life sciences ☐ Behavioural & social sciences ☐ Ecological, evolutionary & environmental sciences

For a reference copy of the document with all sections, see nature.com/authors/policies/ReportingSummary-flat.pdf

Life sciences study design

All studies must disclose on these points even when the disclosure is negative.

Sample size	Sample sizes were determined based on the availability of deceased ROS and MAP participants at time of study conception. They were not determined a priori using power calculations, as data collection for these studies (as well as the IMAS study in which we were able to extend our genetic effects to in vivo microglial activation using PET imaging) is part of ongoing grants. As such, our analyses constituted secondary analyses of these data, though future processing of samples for follow-up study is a priority given the strength of our findings.
Data exclusions	Subjects with missing genotype data that could not be adequately imputed by the Michigan Imputation Server were flagged during quality control and excluded from analyses. In total, 26 out of 2093 genotyped subjects were excluded. This exclusion resulted from large missing blocks of genotypes and thus these 26 subjects were never eligible for inclusion in genetic analyses.
Replication	Rigorous model validation was performed to achieve conservative estimates of effect sizes of PAM on pathological AD. Binay outcome model AUCs are reported after 1000 iterations of bootstrapping, and subsequent models of continuous outcomes were evaluated using robust statistics. As noted in the manuscript, direct replication of our genetic findings is not possible due to the lack of comparable samples. However, we believe that our genetic analysis of in vivo TSPO PET imaging helps validate our observed chromosome 1 association with PAM.
Randomization	This was a retrospective analyses of cohort study data attained as part of larger, ongoing projects. As such, no a priori randomization or grouping of experimental variables was performed. One aspect of our analyses - polygenic associations of traits with PAM, based on summary statistics - relies on assumptions of Mendelian randomization, which is an observed property of allelic assortment in populations, a natural phenomenon that "randomizes" individuals to their genotypic groups.
Blinding	Neuropathologists were blinded to all clinical data prior to autopsy. All clinical, 'omic, and neuropathological data was de-identified prior to analysis. While we recognize the inherent identifiable properties of this type of detailed biological data, no attempt was made at any point to reconstruct identities or biological relationships (beyond ensuring no familial confounding) among study participants.

Reporting for specific materials, systems and methods

Materials & experimental systems

n/a	Involved in the study
☒	☐ Unique biological materials
☐	☒ Antibodies
☒	☐ Eukaryotic cell lines
☒	☐ Palaeontology
☒	☐ Animals and other organisms
☐	☒ Human research participants

Methods

n/a	Involved in the study
☒	☐ ChIP-seq
☒	☐ Flow cytometry
☒	☐ MRI-based neuroimaging

Antibodies

Antibodies used	anti-human HLA-DP, DQ, DR antibodies (clone CR3/43; DakoCytomation, Carpinteria CA; 1:100)
Validation	Catalog #: MA1-25914 . From data sheets on thermofisher.com: "detects HLA DR + DP + DQ from human samples. MA1-25914 has been successfully used in immunohistochemistry (paraffin) procedures. The MA1-25914 immunogen is considered to be commercially sensitive." Three publications are cited on the fisherthermo website (Wang et al., 2016, Clinical Cancer Research; Wick et al., 2014, Clinical Cancer Research; Milne et al., 2008, PLOS ONE). In addition our group has published data using this antibody: Bradshaw et al., 2013, Nature Neuroscience.

Human research participants

Policy information about studies involving human research participants

Population characteristics	The ROS/MAP studies are longitudinal cohort studies of two different populations: religious community of brothers, nuns, sisters, and priests (ROS); and the general population of elderly (MAP). All individuals are age 65 or older at time of study entry and free of dementia. As such, there is a survival bias inherent in the design (only individuals living to 65 without dementia are included). Also, there are uniquely resilient properties, mostly having to do with lifestyle, present in the ROS study. In our analyses of microglial density, all subjects were from MAP, so this characteristic does not affect our results or their interpretation.
Recruitment	Participants of MAP are recruited from retirement communities throughout northeastern Illinois; the only exclusion criteria is the inability to sign the Anatomical Gift Act, and because all clinical evaluations are performed as home visits, co-morbidities common in population-based epidemiologic studies are well represented, reducing the “healthy volunteer effect” seen in many cohort studies.
